# Chair of Surgery in the University of Glasgow

**Published:** 1860-05

**Authors:** 


CHAIR OF SURGERY IN THE UNIVERSITY OF GLASGOW.—The chair of surgery in this ancient institution, rendered vacant by the death of the lamented Lawrie, and temporarily filled, after the demise of that distinguished gentleman, by Dr. George H. B. Macleod, who earned so much reputation in the East, and who is so well known among us by his "Notes on the Surgery of the Crimean War," has been conferred upon Mr. Lister, of Edinburgh, a son-in-law of Mr. Syme. The defeat of Mr. Macleod, who was so admirably qualified for the position, was occasioned on political grounds. Mr. Lister is said to be a young man of high professional attainment and promise.

